# Synergistic properties of cellulases from *Clostridium cellulovorans* in the presence of cellobiose

**DOI:** 10.1186/s13568-015-0169-5

**Published:** 2016-01-04

**Authors:** Kosuke Yamamoto, Yutaka Tamaru

**Affiliations:** Department of Life Sciences, Graduate School of Bioresources, Mie University, 1577 Kurimamachiya, Tsu, Mie 514-8507 Japan; Department of Bioinfomatics, Mie University Life Science Research Center, 1577 Kurimamachiya, Tsu, Mie 514-8507 Japan; Laboratory of Applied Biotechnology, Mie University Industrial Technology Innovation Institute, 1577 Kurimamachiya, Tsu, Mie 514-8507 Japan

**Keywords:** *Clostridium cellulovorans*, Glycoside hydrolase family 9, Glycoside hydrolase family 48, Synergistic effect, Product inhibition

## Abstract

An anaerobic mesophile, *Clostridium cellulovorans*, produces a multienzyme complex called the cellulosome and actively degrades polysaccharides in the plant cell wall. *C. cellulovorans* also changes cellulosomal subunits to form highly active combinations dependent on the carbon substrate. A previous study reported on the synergistic effects of exoglucanase S (ExgS) and endoglucanase H (EngH) that are classified into the glycosyl hydrolase (GH) families 48, and 9, respectively. In this study, we investigated synergistic effects of ExgS and EngK, a GH9 cellulase different from EngH. In addition, since EngK was known to produce cellobiose as its main product, the inhibition on cellulase activity of EngK with cellobiose was examined. As a result, the effect of cellobiose inhibition on EngK coexistent with ExgS was found to be much lower than that with EngH. Thus, although EngH and EngK are in the same GH9 family, enzymatic activity in the presence of cellobiose was significantly different.

## Introduction

**Fig. 1:**
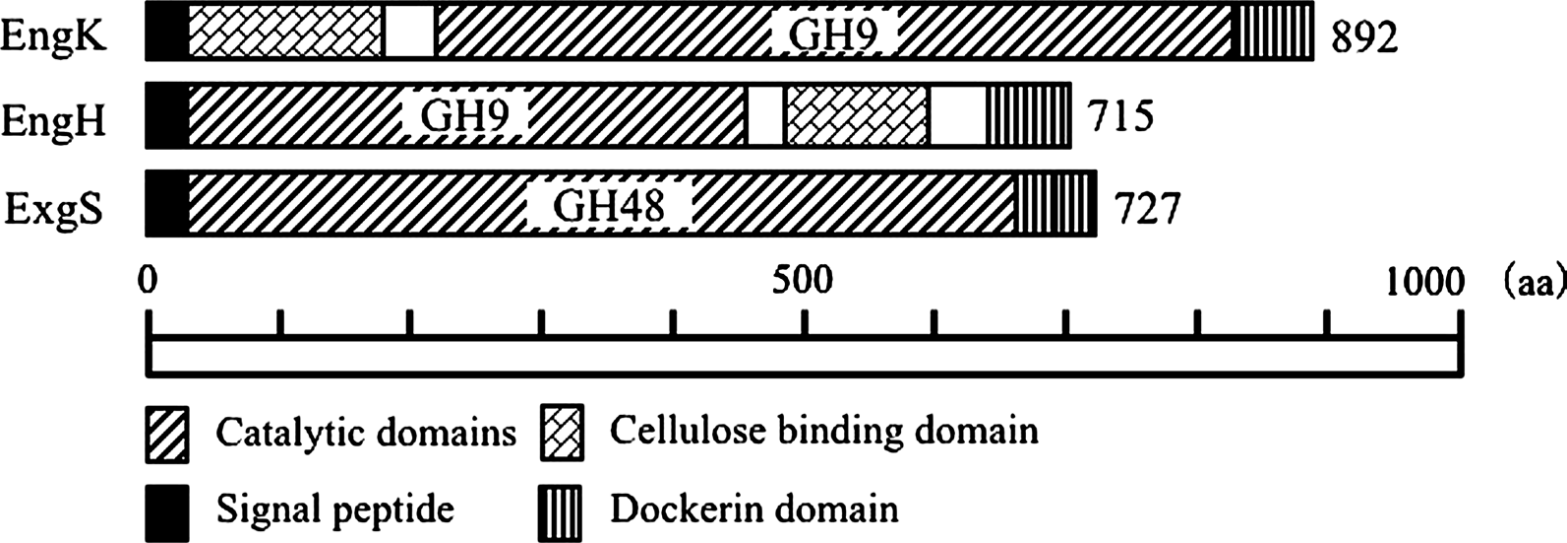
Schematic models for EngK, EngH and ExgS from *C. cellulovorans*. *Numbers* in the schematic models indicate glycoside hydrolase (GH) family. Protein names (Eng) and the length of amino acid sequence (aa) are represented on the *left* and *right sides*, respectively, of the models

*C. cellulovorans*, an anaerobic mesophile, efficiently degrades polysaccharides by producing an extracellular multienzyme complex called the cellulosome (Doi and Tamaru 2001; Doi et al. 1994; Goldstein et al. 1993; Shoseyov et al. 1992; Sleat et al. 1984; Takagi et al. 1993). Previous study reported that a cellulosomal gene cluster *cbpA*-*exgS*-*engH*-*engK*-*hbpA*-*engL*-*manA*-*engM*-*engN* was found in the *C. cellulovorans* genome (Tamaru et al. 2000; Tamaru et al. 2010). Additionally, EngE belonging to glycosyl hydrolase (GH) family 5 plays important roles on cellulose degradation (Shoseyov and Doi 1990; Tamaru and Doi 1999). A previous genome sequence analysis revealed that this organism has genes encoding 17 cellulosomal cellulases, 10 cellulosomal hemicellulases and 63 non-cellulosomal enzymes related to degradation of polysaccharides such as cellulases and hemicellulases, but also pectinases (Doi et al. 1998; Tamaru et al. 2010, 2011). An expression pattern of polysaccharolytic enzymes was changed for degradation of each carbon source (Han et al. 2003, 2004; Han et al. 2005; Yamamoto and Tamaru. 2014). However, proteomic analysis reported that EngH (GH9), EngK (GH9) and ExgS (GH48) were produced rather abundantly and consistently irrespective of the type of growth substrates (Fig. 1, Matsui et al. 2013; Morisaka et al. 2012). These results indicated that these enzymes played a critical role on cellulose degradation. On the other hand, the properties of cellulosomal family 9 cellulases EngH, EngK, EngL, EngM, and EngY in *C. cellulovorans* were analyzed in previous studies (Arai et al. 2006). These studies showed that cellulosomal family 9 cellulases had different activities against various cellulases such as carboxymethyl cellulose (CMC) and crystalline cellulose (Avicel), although they are all classified as GH9. Synergistic effects of cellulosomal subunits EngH and ExgS were studied (Murashima et al. 2002). However, the synergistic effects of EngK and ExgS have not been studied as yet.

In this study, the synergistic effects of ExgS (GH48) and EngK (GH9) or EngH (GH9) in the presence of cellobiose were compared.

## Methods

### Bacterial strains and media

*C. cellulovorans* (ATCC 35296) was used as the source of chromosomal DNA. *Escherichia coli* HST08 (TaKaRa) and origami (Novagene) were used for the construction of plasmids and cloning host for the production of recombinant proteins, respectively.

### Plasmid construction and expression of recombinant proteins

Recombinant EngK, ExgS, and EngH were expressed with the pCold-I (TaKaRa) vector and pCold-TF vector (TaKaRa), respectively. DNA fragments encoding each gene were amplified by polymerase chain reaction from the *C. cellulovorans* chromosomal DNA with the primers containing restriction sites (Table 1). The amplified PCR fragments were digested with restriction enzymes and inserted into pCold-I or pCold-TF digested with the same pair of restriction enzymes to generate pCold-I-EngK, pCold-I-ExgS and pCold-TF-EngH. *E. coli* origami harboring pCold-I-EngK, pCold-I-ExgS and pCold-TF-EngH were grown at 37 °C in Luria–Bertani medium supplemented with ampicillin (100 µg/ml) to an optimal density at 600 nm of 0.4–0.5. The culture was supplemented with a final concentration of 0.5 mM isopropyl-ß-d-thiogalactoside (IPTG) and growth continued at 15 °C for 24 h. The culture was refrigerated at 15 °C quickly and left to stand for 30 min.

**Table 1:** Designs of primers used in this study

Primer	Sequence	Restriction site	Prasmid
engH-TF-F	GTTCTCGAGTTATCAGGAATCTTGGGTGCAACTTC	*Xho*I	pCold-TF-engH
engH-TF R	TTAGGATCCCTGATAAAAGTAG	*BamH*I	pCold-TF-engH
sacI-engK	TTGAGCTCATGCGTAGTAAAAAATTAATAGCTTG	*Sac*I	pCold-I-engK
engK-xhoI	CCCCTCGAGTTAAGAAAGAAGTTTCTTCT	*Xho*I	pCold-I-engK
sacI-exgS	GGGAGCTCATGAGAAAAAGATTAAATAAGATCGTTG	*Sac*I	pCold-I-exgS
exgS-xhoI	CCCCTCGAGTTAAGCAAGAAGTGCTTTCT	*Xho*I	pCold-I-exgS

### Purification of recombinant proteins

The cultured *E. coli* cells were harvested by centrifugation, and were washed and disrupted by sonication. Cell debris was removed by centrifugation. The cell-free extracts were centrifuged (for 30 min at 4 °C at 20,000*g*) and separated from the supernatant and the pellets, respectively. TF-EngH was purified from the supernatant. EngK and ExgS were purified from the pellets. The supernatant (for TF-EngH) was applied onto HisTrap HP (GE healthcare) and eluted by 20 mM phosphate buffer (pH 7.4) containing 500 mM NaCl and 500 mM imidazole. The trigger-factor (TF) tag was removed from TF-EngH by HRV-3C protease (Novagen). The pellets (for EngK or ExgS) were solubilized with 8 M urea and renatured essentially as described previously (Liu and Doi 1998). The purified enzymes containing the fractions were dialyzed against 50 mM acetate buffer (pH 6.0). The concentration of purified proteins was measured by protein assay kit from Bio-Rad, using bovine serum albumin as the standard.

### Enzyme assay

Enzyme activities were assayed in the presence of 0.5 % (wt/vol) concentration of acid-swollen cellulose at 37 °C in 50 mM acetate buffer (pH 6.3) containing 2.5 mM CaCl_2_, 0.08 mg/ml tetracycline and 0.06 mg/ml cycloheximide. Final enzyme concentration was prepared at 20 nmol/ml. Samples were collected and immediately boiled for inactivation of the enzymes. 5 or 10 mg/ml of cellobiose were added to the enzyme assay mixture for inhibition of synergistic activities among EngH, EngK, and ExgS. The reducing sugars were determined by the DNS method, as d-glucose equivalents. Activities were expressed in units, 1 U defined as the amount of enzyme releasing 1 µmol of reducing sugar per min.

## Results

### Synergy effect on acid swollen cellulose between recombinant proteins EngK, EngH and ExgS

**Fig. 2:**
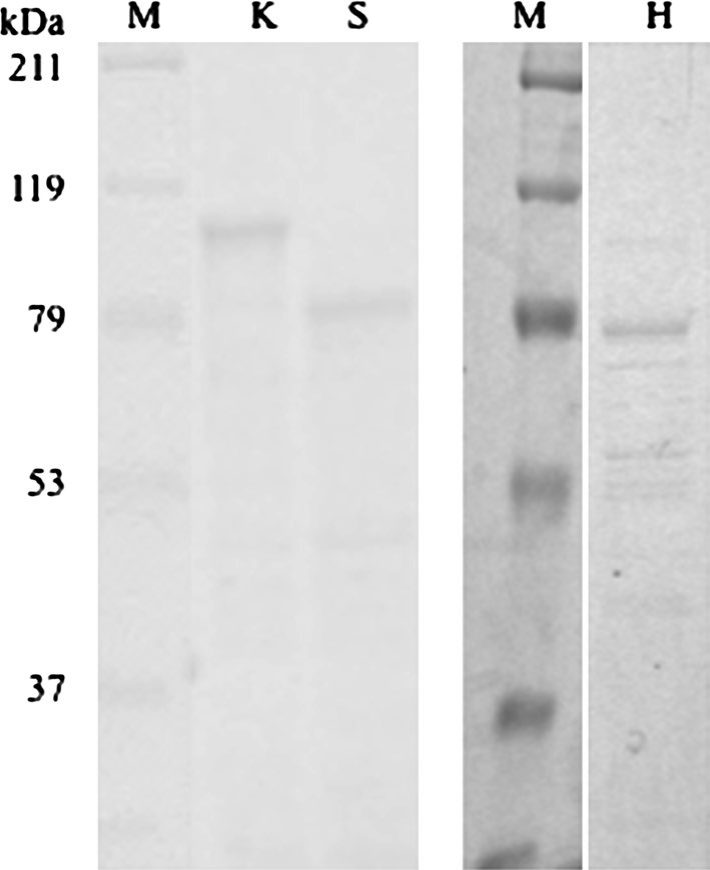
SDS-PAGE of the purified recombinant enzymes. The gel was stained with Coomassie brilliant blue R-250. *Lanes M* protein molecular mass standard; *lane K* EngK; *lane S* ExgS; *lane H* EngH

**Fig. 3:**
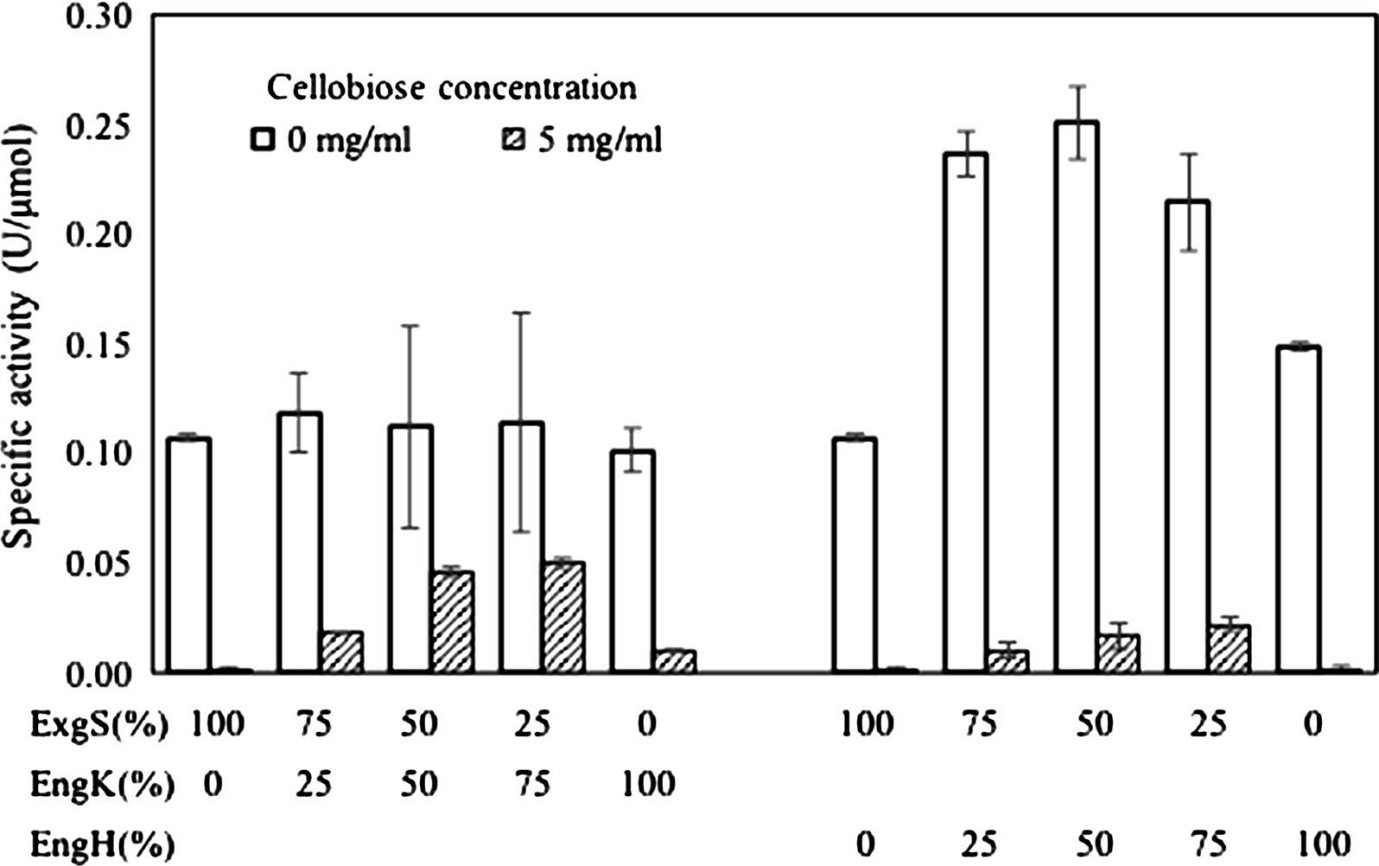
Specific activities of recombinant EngK and ExgS against acid-swollen cellulose. Two cellulosomal subunits were mixed at various compositions as shown in the X axes. The total concentration of enzymes was fixed at 20 nmol/ml

Purification of individual recombinant enzymes, EngH, EngK and ExgS is shown in Fig. 2. Figure 3 and Table 2 show the synergy effects on activities against acid-swollen cellulose between the recombinant enzymes among EngH, EngK and ExgS. Specific activities of ExgS, EngK and EngH were 0.107, 0.102 and 0.149, respectively. As previous studies, the synergistic effect between ExgS and EngH was detected. The mixture of the recombinant enzymes of ExgS and EngH showed the highest specific activity (0.251 U/μmol) at a molar ratio of ExgS to EngH of 50:50 %. On the other hand, the mixture of the recombinant enzymes of ExgS and EngK showed specific activity of only 0.119 U/μmol at the most (molar ratio of ExgS to EngK of 75:25 %).

**Table 2:** Synergy degrees and the inhibition of cellulases activity on cellulose by cellobiose

Molar percentage of enzyme (%)			Synergy degree^a^	Inhibition rate (%)
ExgS	EngK	EngH		
100	–	–	–	98.5
75	25	–	1.120	84.8
50	50	–	1.073	58.9
25	75	–	0.824	56.1
–	100	–	–	90.1
75	–	25	2.011	95.6
50	–	50	1.958	93.4
25	–	75	1.549	89.7
–	–	100	–	98.7

^a^The synergy degrees are shown as the actual activities divided by the summation of each cellulases activity

### Inhibition of synergistic activities among EngH, EngK, and ExgS by cellobiose

Enzymatic activities of all recombinant enzymes and their synergistic activities were inhibited by 5 mg/ml cellobiose (Fig. 3; Table 2). The inhibition rates of ExgS, EngH or EngH were 98.5, 90.1 or 98.7 %, respectively. The highest specific activity of the mixture of EngH and ExgS was 0.251. The activity was inhibited to 0.017, that is, the inhibition rate was 93.4 %. In contrast, the inhibition rate of the mixture of EngK and ExgS was 56.1 %, when the molar ratio of EngH to ExgS was 25:75 %. The synergistic activity of EngK and ExgS containing 5 mg/ml cellobiose was more than twice the synergistic activity of EngH and ExgS. No activities were detected in each reaction mixture in presence of 10 mg/ml cellobiose.

## Discussion

Synergistic effects with either EngK or EngH and ExgS were detected in the assay against acid-swollen cellulose (Fig. 3; Table 2). These synergies were lower than the synergy between EngH and ExgS that has been reported in a previous study (Murashima et al. 2002). In addition, the inhibition of synergistic effect by cellobiose was different between EngH and EngK (Fig. 3; Table 2). The inhibition of EngK with ExgS by cellobiose was lower that of EngH with ExgS. These results indicated that the difference between EngH and EngK is not only with their enzymatic properties but also with their synergistic effects.

**Fig. 4:**
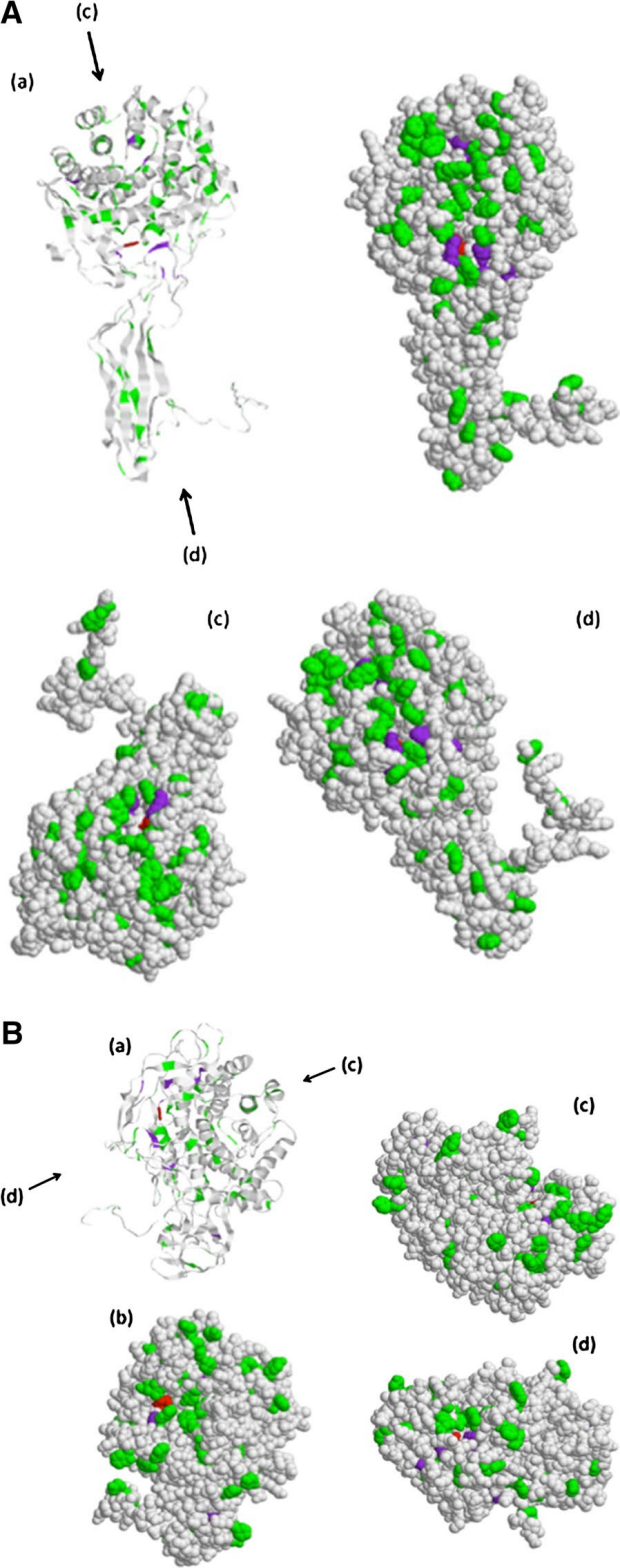
The difference of cleft shapes between EngH (**A**) and EngK (**B**). Three-dimensional models for EngH and EngK based on homologues of known structure were built by 3D-JIGSAW. The models were visualized using Rasmol tool. The aromatic amino acids, histidine and putative catalytic base in the models of the ribbon diagram (*a*) and the space-filling (*b*, *c*, *d*) were colored *green*, *purple* and *red*, respectively. The *arrows* indicate the direction of looking in the cleft

EngK has enzymatic activity against cellotriose (Arai et al. 2006). *R. cellulolyticum* Cel9E (GH9) can cleave cellotriose, cellotetraose and cellopentaose to cellotriose, cellobiose and glucose (Gaudin et al. 2000). In particular, cellobiose constitutes more than 90 % of products when Cel9E cleaves Avicel (Ravachol et al. 2014) or amorphous cellulose (Gaudin et al. 2000). In addition, the crystalline structure of Cel9G has already been revealed; EngK has fewer aromatic residues than Cel9G (Mandelman et al. 2003). For this reason, it appears that cellobiose does not remain for a long time in the active-site cleft, and oligosaccharides can easily fit with the active-site cleft even in the presence of cellobiose. Furthermore, the previous study indicated that EngK does not produce oligosaccharides longer than cellotriose (Arai et al. 2006). Identity of amino acid sequences between EngK and EngH was low, whereas identity of amino acid sequences between Cel9G and EngH was high. Furthermore, enzymatic properties of EngK and EngH were quite different. These differences would be revealed by crystal structure analysis of EngK, and particularly by co-crystallization EngK and cellobiose. Three-dimensional models for EngH and EngK based on homologues of known structure also would help in these predictions. According to the models, the cleft of EngK was shorter than the cleft of EngK. In EngH and EngK, the number of aromatic amino acids, and histidine in the putative active site cleft of EngH and EngK were seven, two and six, one, respectively (Fig. 4).

The importance of EngE (GH5), ExgS (GH48) and EngH (GH9) which are main subunits in the *C. cellulovorans* cellulosome has been reported by a number of studies. Synergistic effects between those cellulases were demonstrated by many enzymatic studies. Some of these studies have found that the enzymatic property of the cellulosome changes depending on the subunit composition of the cellulosome. On the other hand, new insights of synergistic effects between EngK (GH9) and ExgS under the inhibition by cellobiose were shown in this study. Complexation of cellulosomal enzymes perhaps change their inhibition by cellobiose. These results supported previous studies on the cellulosome of *C. cellulovorans* and the other clostridia.
